# Summary of the proceedings of the International Forum 2019: “What are the strategies to engage the young generation”

**DOI:** 10.1186/s13244-019-0827-2

**Published:** 2020-01-23

**Authors:** European Society of Radiology (ESR)

**Affiliations:** Am Gestade 1, 1010 Vienna, Austria

**Keywords:** Young generation, Strategies, Radiology, Future

## Abstract

The ESR International Forum at the ECR 2019 discussed strategies for engaging the young generation. The future of radiology is a crucial topic that goes hand in hand with tomorrow’s radiologists. The European Society of Radiology (ESR) established the ESR International Forum in order to discuss hot topics in the profession of radiology with non-European radiological partner societies. At the ESR International Forum 2019, different strategies, initiatives and ideas were presented by the different societies with regard to engaging young doctors into radiology.

## Main messages

- It is important to understand the characteristics of the younger generation and develop a new approach
- Younger generation needs to be guided, mentored and delegated more responsibilities
- New and innovative forms of engagement through technology need to be adopted
- Radiologists’ role is changing, and the image of a radiologist’s job accordingly needs to be re-envisioned
- Increased use of social media and technology is the crucial element for engaging the younger generation

## Introduction

The ESR International Forum was established by the European Society of Radiology (ESR) with the aim of discussing pivotal themes in the field of radiology with international societies from outside Europe. The ESR International Forum is held every year at the European Congress of Radiology (ECR) and participation is by invitation only. Previous topics discussed are the relation between radiology and nuclear medicine, the position of ultrasound in radiology, the relation of general radiology and subspecialty radiology, the implementation of clinical decision support and imaging referral guidelines in the clinical routine, the position of interventional radiology within radiology, and value-based radiology.

The following societies were invited to deliver a presentation and to present the point of view of their respective country or region: The American College of Radiology (ACR), the Asian Oceanian Society of Radiology (AOSR), the Brazilian College of Radiology (CBR), the Canadian Association of Radiologists (CAR), the Chinese Society of Radiology (CSR), the Colombian Association of Radiology (ACR), the Egyptian Society of Radiology and Nuclear Medicine (ESRNM), the Indian Radiological and Imaging Association (IRIA), the Inter-American College of Radiology (CIR), the International Society of Radiology (ISR), the Japan Radiological Society (JRS), the Korean Society of Radiology (KSR), the Mexican Federation of Radiology and Imaging (FMRI), the Mexican Society of Radiology and Imaging (SMRI), the Radiological Society of North America (RSNA), the Radiological Society of Saudi Arabia (RSSA), the Radiological Society of Thailand (RST), the Royal Australian and New Zealand College of Radiologists (RANZCR), the Royal College of Radiologists of Thailand (RCRT), and the Radiological and Diagnostic Imaging Society of Sao Paulo (SPR). The host presented the situation in Europe. In addition, representatives of non-European past “ESR meets” countries of the last five years were invited to the meeting.

### The situation in North America

E. Lee presented on behalf of the **Canadian Association of Radiologists (CAR)**. Placing special emphasis on engagement as the main task of any association, E. Lee pointed out that in a landscape that is increasingly competitive, it is important to demonstrate value and appeal to current and future members. Historically, radiology has been considered one of the most competitive specialties in the Canadian Resident Matching Service (CaRMS). In recent years there has been widespread speculation that applications are decreasing as the number of radiology positions has increased while number of applicants has not grown commensurately. The situation has, however, mostly corrected itself. Currently around 80% of Canadian radiologists are members of the CAR. Seeing how the constant increase of membership is vital for the healthy functioning of the association, the CAR has set a goal of 2% increase in membership per year, with the increase in 2018 being 10%. In order to achieve this goal CAR is strongly focusing on residents, while maintaining relationships with reciprocal provinces and developing new partnerships with non-reciprocal provinces. In order to tackle the challenge of motivating resident members the CAR has implemented a strategy targeted specifically at them, such as a dedicated resident section on the CAR website, discounted membership and conference registration, and a Membership Working Group, among others. E. Lee explained that the idea behind the strategy is that the chances of retention are significantly increased if members can be recruited before they enter the workforce. The CAR emphasises word of mouth and testimonials as one of the most important communication techniques. According to E. Lee, having ambassadors spread the word will be the single most effective method in reaching the association’s recruitment and retention goals. Vital elements of this approach are identifying key opinion leaders that can speak the key messages, liaising with non-members, providing a visibility platform and empowering ambassadors with tools and resources. Finally, E. Lee outlined significant opportunities for internationals. The CAR can help bridge the connection and provide information on placements and jobs for new graduates as well as offer a discounted rate for international members as an excellent way of getting Canadian specific information.

V. P. Jackson, presenting on behalf of the **Radiological Society of North America (RSNA)**, explained that encouraging young people to join and become members is a high priority. The societies are competing for members’ time and money. That is why it is important to understand what young people want and need, as opposed to what the societies think they need. In order to tackle this issue, the RSNA has developed a variety of tools, including free membership (includes RSNA Annual Meeting and 5 journals), volunteer committee service opportunities, a specialised Resident and Fellow Committee, introduction to Research programme, R&E Foundation grants, as well as the RSNA Diagnosis Live gaming tool that is free for residency programs worldwide. The RSNA gives special attention to early-career practitioners. They do not pay full dues during the early years of membership but nevertheless have full access to the RSNA Annual Meeting and journals, volunteer service opportunities, education for all aspects of any type of career as well as R&E Foundation grants.

- G. McGinty, on behalf of the **American College of Radiology (ACR),** emphasised the incredibly vibrant resident and fellow section of the ACR. The ACR provides free membership to residents and fellows and offers representative governance to both trainees and young practicing physicians including a seat on the ACR board for this latter group. This representation allows residents and fellows to bring policy resolutions to the ACR Council floor, thus effectively highlighting topics that are pertinent to this community. An important activity mentioned by G. McGinty is the ACR community’s active engagement on social media as well as its internal networking platform called Engage. This online conversation helps identify concerns among the trainee and young physician groups and helps set priorities for the organisation as a whole.

### The situation in Latin America

M. Hernandez Cruz, on behalf of the **Mexican Federation of Radiology and Imaging (FMRI),** presented the current situation in Mexico. Due to the fact that only around 50% of the 164 medical schools and faculties in Mexico include radiology in their curriculum, the students have little knowledge and interest in radiology. That is why the FMRI strives to generate interest among medical students. In order to achieve their goals, the FMRI has asked the Secretariat of Public Education to include radiology in all their programmes. The FMRI also provides teaching tools and the newest technologies to faculties and schools and actively involves students in the learning process. M. Hernandez Cruz further provided an example of a successful case study from the Universidad Autonoma de Nuevo Leon. This university includes radiology in its program and offers students with the best results the opportunity to become instructors after successfully finishing a course, thus giving the students an active role in the teaching-learning process. This approach has resulted in an increased interest in radiology and its role in patient care. Additionally, 118 students have so far took part in the radiology course as instructors, more than 50 applicants per year have registered for the program and the overall success of the students in the medical school has increased. Finally, M. Hernandez Cruz summarised the strategy for demonstrating medical students the importance of radiology. He emphasised the need for image in patient care, leadership in healthcare and innovation and technology, pointing out that radiologists must see new technologies as an opportunity. In conclusion, M. Hernandez Cruz expressed positivity regarding the future of radiology saying that radiology will continue to be an important part of medicine.

M. Palacios Montesinos on behalf of the **Mexican Society of Radiology and Imaging (SMRI)**, spoke about the importance of understanding millennials as the new generation. According to M. Palacios Montesinos, regardless of their nationality millennials have some common traits, such as being digital natives and multitasking among others. The societies need to adopt new programmes, motivate and encourage millennials, and develop specialised plans tailored to each generation. M. Palacios Montesinos presented three main strategies of the SMRI. Firstly, it is important to spread enthusiasm, with a dose of reality, to better understand technological processes and to encourage and guide young people. Furthermore, activities aimed at the younger generation need to be posed as challenges or goals and it is crucial to dedicate time and effort to obtain positive results. Finally, investments in communication technologies and the promotion of virtual training tools, technological advances and the A.I. are crucial. M. Palacios Montesinos concluded that the historical, political, social, cultural and technological changes have marked and moulded the millennials in different ways and that their role and generational importance will depend on their way of engaging with the world.

M. E. Oyuela representing the **Colombian Association of Radiology (ACR)**, presented the context of radiology education in Colombia. The country operates 18 radiology post-graduate programmes, with 265 residents currently in the ACR. Since 2017 the ACR established quality standards programme for radiologist in training. The programme serves as a reference for universities’ self-assessment of training programmes and allows them to develop new education curricula. M. E. Oyuela pointed out that in order to engage new generations it is crucial to identify radiologists’ mental barriers. Having those barriers in mind, the ACR has developed a strategy proposal divided into two aspects: technological/academic and humanistic. From the technological and academic point of view, M. E. Oyuela emphasised the importance of training with a systematic approach, allocating time and resources for research and greater involvement of mentors. Online radiological networks, web-based learning, social media as well as delegating residents responsibilities in accordance with their competences present important aspects of the ACR strategy. From the humanistic point of view, important elements include person centred care, communication skills, work in interdisciplinary groups, professional and ethical competence, greater work-life integration, equality of opportunity regardless of gender, emotional support, empathy and respect. Finally, M. E. Oyuela stressed the importance of minimally invasive procedures, promotion of accredited services, adapting to change and participation in the society’s activities. M. E. Oyuela concluded that these strategies will contribute to the engagement of new generations of young radiologists, and ultimately lead to better professionals and better human beings.

A. Sarmet Santos, on behalf of the **Brazilian College of Radiology (CBR)**, presented the background information on the society as the official national entity representing the radiologist in Brazil. The society currently has more than 12,000 radiologists, with around 40% younger than 40 years. There are currently 212 centres for training in radiology in Brazil with 2,212 residents in training. A. Sarmet Santos pointed out the importance of understanding what young people expect form the societies - learning and professional formation, protection of their interest and opportunities other than just a job. In that sense the CBR organises the annual Brazilian Congress of Radiology, continuous medical educational courses, annual ESOR courses as well as hands-on courses. A. Sarmet Santos further pointed out that the CBR publishes formal job opportunities in every communication outlet of the CBR and offers special awards to residents. The CBR highlights opportunities for clinical and research fellowships for young professionals, within and outside the country, by consulting a network of established academic radiologists. Furthermore, the CBR has an agreement with the Brazilian Ministry of Education aimed at supervising and preforming regular evaluation of every residence program in the country. The CBR also acts in cooperation with the Brazilian Medical Association and Federal Council of Medicine to ensure a reliable radiology practice for all professionals, especially the new ones, and offers general legal counselling to every member of the CBR. A. Sarmet Santos emphasised the importance of mobile learning initiatives and other educational initiatives such as Radiologia Brasiliera journal, open access journal, hands-on training and face-to-face training series. Finally, A. Sarmet Santos presented the CBR goals for the 2019-2020 period, primarily the launch of the CBR’s Virtual Learning environment, that will offer distance courses and performance evaluation.

C. Homsi, represented the **Radiological and Diagnostic Imaging Society of Sao Paulo (SPR)**. The SPR is a society of more than 7,000 members, with around 50% younger than 34 years. The SPR offers these young professionals several benefits that provide a high level of educational content and opportunities for professional improvement. C. Homsi pointed out the online course for residents that is free of charge for members of the SPR and content-wise provides all the information that a resident should know at the end of the 3-year programme of residency. Another important tool of the SPR is the Feres Secaf course, a 4-day event with 180 lecturers and more than 1,800 participants. The course serves as the annual residents meeting and its scientific program offers themes regarding daily routine of diagnostic imaging, professionalism courses as well as case review discussions. C. Homsi further mentioned the activities of the Roentgen Club and the Manoel de Abreu Club that offer lecture presentations and case-based review discussions with cases being prepared and presented by the residents. The SPR maintains 15 subspecialist study groups with more than 4,000 participants in 2018, as well as subspecialist advanced courses. The SPR also invests in the hybrid teaching model, a program that starts with an online theoretical phase and then develops the practical module. C. Homsi pointed out that all the members of the SPR have free access to the STATdx platform, the world's largest digital radiology database and that the SPR offers opportunities for residents to participate in international fellowships programs, international congresses and international courses. He concluded that the SPR is very successful in attracting young radiologists and that their main concerns currently are the specialist over 46 years of age that are losing their interest in radiology.

H. Carrete Junior, representing the **Interamerican College of Radiology (CIR)**, gave an overview of the CIR that was founded in 1943. He pointed out the importance of defining the generations properly, making a distinction between millennials, born between 1981 and 1996, who are now residents and radiologists, and post-millennials, born from 1997 onward, who are currently students. As each generation has its own characteristics H. Carrete Junior presented the key factors that in his view differentiate these two groups and suggested adopting a special approach towards the Gen Z as they are the first truly digitally native generation that is accustomed to having information at their fingertips. H. Carrete Junior therefore suggests adopting new strategies for teaching the Generation Z focusing on short lectures with graphical and digestible content, maintaining relevance by explain upfront why a lesson is needed and how it can be applied in the real world as well as establishing student alliances. When it comes to residents and young radiologists H. Carrete stressed the importance of implementing a variety of teaching methods focused on innovation and the issue rather than the organisation. Ultimately, it is important to promote competitions or challenges and demonstrate social responsibility.

### The situation in Egypt

T. El-Diasty, on behalf of the **Egyptian Society of Radiology and Nuclear Medicine (ESRNM)**, spoke about the situation in Egypt. He presented the main problems faced as well as the strategies implemented by the ESRNM to overcome them. Lack of an applicable plan for continued education leads to the young generation not being aware of how the learning process is going to continue. The ESRNM thus proposes setting up a plan with clear methods of application, instructing the young generation and setting up orientation meetings to be done by the training committees all over Egypt. One of the problems faced by the younger generations is that professional training using advanced tools is very localised and limited to certain hospitals due to a relatively low number of such tools in comparison to the number of radiologists. The ESRNM proposed designing a clear clinical training programme that facilitates rotation of the junior radiologists in well-equipped hospitals. T. El-Diasty also spoke about the difficulties faced by the younger generation regarding external training. Very few opportunities for joining western radiology fellowship programmes are available to Egyptian radiologists. The ESRNM therefore advocates for a more active involvement of sponsors and cooperation with international societies/organisations. An important issue is that research is not widely well implemented in universities, teaching hospitals and governmental hospitals. As much as 70% of radiologists in Egypt have not been engaged in any research activity due to limited capacity or lack of mentoring and motivation. The proposed solutions aim to create research teams that offer research courses, provide a research plan to each group of hospitals based on their needs and location and to engage radiologists. Additionally, the ESRNM offers lectures, courses and workshops on scientific writing at different radiology departments and annual conferences and promotes publications through international journals including the Egyptian Journal of Radiology and Nuclear Medicine (EJRNM). Finally, T. El-Diasty pointed out that most of the young generation is not aware of membership benefits. He concluded that the younger generation should be offered free membership, free/reduced registration for training courses/national and international meetings, educational materials, and membership in other societies.

### The situation in Saudi Arabia

L. G. Jamjoom, on behalf of the **Radiological Society of Saudi Arabia (RSSA)**, presented the demographic background of the country emphasising the strength of a very young, well-educated population. L. G. Jamjoom explained that leaders from every area of the society have created an action plan to be completed by 2030 with three fundamental objectives being a vibrant society, a thriving economy and an ambitious nation. In the context of education, and in particular medical education, L. G. Jamjoom pointed out that radiology is integrated in every module throughout the 3rd year of medical school education with medical students additionally attending an intensive 2 week radiology course during the 4th and the 6th year, with lectures in all radiology subspecialties, practical rounds and students’ presentations. Radiology department at the King Abdulaziz University Hospital (KAUH) hosts approximately 10 to 12 interns every month, and the interns get the chance to shadow the residents and attend lectures and departmental meetings. When it comes to residents, there are currently 40 residency programs with a total of 547 residents. Approximately 20 years ago, the government has created a very successful scholarship programme; 90% of Saudi board-certified radiologists complete the fellowship training, with 70% of them doing the fellowship training abroad. The country operates 7 body imaging and non-vascular intervention fellowship programmes and 8 interventional radiology fellowship programmes. In conclusion, L. G. Jamjoom stated that the RSSA plays an active role in education through providing visiting professors for different regions as well as through international collaborations.

### The situation in India

H. Patel, on behalf of the **Indian Radiological and Imaging Association (IRIA)**, presented the situation in India. Radiology is one of the most sought-after specialties in India for more than a decade. More than 50% of radiologists in India are younger than 42. In order to identify the main needs of the younger generation, the IRIA conducted a survey, resulting in a list of priorities and consequently appropriate actions and solutions. Some of the needs expressed by the younger generation include, inter alia, learning for everyone, the use of technological advances such as 3D printing and molecular imaging, increased use of web and social media, as well as more financial support for innovative ideas. In order to tackle these issues, the IRIA has developed a variety of tools aimed at helping the younger generations and meeting their needs. The IRIA organises CMEs at various state and district levels so that radiology teaching can reach every corner of the country, with this year’s primary focus on Interventional Radiology and Breast Imaging. Due to a large interest of the younger generation in sonography, H. Patel pointed out that the IRIA also organises a SonoSummit, the largest conference focused on sonography in India that has workshops and live demos at the forefront. One of the most important initiatives launched by the IRIA is the Youth Wing of Radiology (YUWA), working exclusively on the welfare of postgraduate residents and young radiologists. As part of its activities in 2019, YUWA celebrated international women’s day in 100 cities in India, organising meetings exclusively for women radiologists and dealing with topics such as women's imaging, finance management and health tips. H. Patel concluded that the YUWA initiative helps understand and address the problems faced by radiology residents and young radiologists, serves as a platform to showcase talent of the younger generation and helps radiology residents by preparing them for future challenges.

### The situation in Thailand

A. Churojana, presenting on behalf of the **Royal College of Radiologists of Thailand (RCRT)** and the **Radiological Society of Thailand (RST),** provided background information regarding radiology training in Thailand. A. Churojana explaining that there are currently 8 institutes that provide training in diagnostical radiology, radiation oncology and nuclear medicine. The quotas for training in diagnostic radiology in 2019 have been filled, indicating interest of the younger generation in this field. However, as it is important to engage the younger generation even further, A. Churojana presented a three-fold approach for achieving that goal, namely undergraduate promotion, postgraduate promotion and public promotion. Undergraduate promotion aims to convey main principles of radiology and image interpretation, provide positive attitude towards radiology and introduce advanced tools. Postgraduate promotion entails a roadshow regarding radiology training, “open house” activities of each institute, radiology outreach meetings/programmes, team building activities in radiology and establishing connection and collaboration between all related radiological societies. Finally, A. Churojana explained the importance of public promotion in Thailand. Public promotion is done through a specialised website aimed at undergraduates that promotes radiology and related fields, explains treatment of diseases and fosters interest in new technologies and tools. A. Churojana concluded that an important part of public promotion is inviting medical students and young radiologists to be part of public promotion teams as well as organising Corporate Social Responsibility (CSR) events.

### The situation in China

Z. Y. Jin, on behalf of the **Chinese Society of Radiology (CSR),** spoke about the situation in China. China currently has around 16,000 radiologists, 30% of which are young radiologists. Z. Y. Jin stressed the importance of understanding the character of the young generation for developing a correct approach. Younger generation is independent, creative, self-confident and energetic. Accordingly, the approach must be flexible and must not involve over-management of the younger generation. Z. Y. Jin pointed out that it is important to create an environment with best modalities, best technologies and best mentors for the young generation and to develop personalised pathways in order to find the right person for the right position. In that sense the CSR provides a lot of opportunities for younger people including standardisation of the fundamental training programmes and diverse training programmes/chances from the academic societies and institutions.

### The situation in Korea and Japan

W. Lee presented on behalf of the **Korean Society of Radiology (KSR).** The action plan of the KSR for engaging the younger generation includes a membership committee, webpage with a member lounge, as well as a specific Korean Congress of Radiology (KCR) event. The webpage provides younger generation with the opportunity to anonymously upload any comment or question that is replied by a KSR officer. Two keywords of the KSR strategy aimed at the younger generation are communication and share of information. A vital aspect of communication is the opportunity to discuss any topic or idea and make inquiries via the website. W. Lee explained that this in turn allows the KSR to identify problems by collecting and categorising data and provide improved service to the young generation based on the collected data. When it comes to the share of information, the KSR makes sure to collect basic member information, develop databases or distribution charts and help the young generation find the information easily. Finally, it is important to engage young generation even more in events such as the KCR. During the KCR, the KSR provides a young radiologists lounge, organises photo contests and movie clips/user-created content (UCC) contests where participants capture the moments of the KCR and have a chance to win awards and prizes.

Y. Imai, on behalf of the **Japan Radiological Society (JRS)**, talked about the situation in Japan. Y. Imai initially presented the strategies for research work pointing out that the JRS offers several awards to young researches, including academic awards, JRS related awards as well JRS related research grants. The Japan Radiological Society also focuses on strategies for medical training abroad fostering exchange programmes such as the Japanese-German Radiological Affiliation and the French Society of Radiology – Japan Radiological Society (SFR-JRS) Fellowship Program Exchange. Furthermore, Y. Imai explained that the most expected development happening in the next generation would be the application of A.I. to radiology with multiple effects. Y. Imai, however, pointed out that recently there has been a false rumour that radiology specialists are one of the jobs to be replaced by the A.I. Consequently, the number of young people who aspire to become radiology specialists is decreasing and there is a fear that people in general will pay less attention to the radiologists’ job. That is why it is of paramount importance to have open lectures and events in order to spread the idea of the importance of the radiologists’ job that includes not only diagnosing the disease, but also selecting an appropriate treatment for each patient. The A.I. is a useful tool for radiologist but ultimately only a radiologist can check whether the A.I. made an error. Accordingly, the JSR is preparing a special corner for the public, including children, at the General Assembly of the Japanese Association of Medical Sciences (JAMS) in order to demonstrate the most recent advancements in radiology.

### The situation in the Asia-Oceania region

D. Varma, on behalf of the **Asian Oceanian Society of Radiology (AOSR)**, presented some background information on the AOSR. As the AOSR assembles different national societies it cannot deal with individual country-specific issues only. That is why the AOSR established the Asian Oceanian School of Radiology (AOSOR). The three major projects of the AOSOR include the AOSOR Conjoint Session, the AOSOR Youth Club and international visiting professors. D. Varma put special emphasis on the AOSOR Youth Club as an important initiative to engage the young generation. The AOSOR Youth Club was established in 2015 with the main aims of providing intensive basic leadership skills, fostering engagement of future AOSR leaders, strengthening inter-society relationship and establishing AOSOR-YC alumni. The Youth Club organises 3-day leadership training courses for around 15 trainees from member societies with tutors sponsored by AOSOR and the host society. D. Varma concluded that this approach provides a different solution for engaging the young generation and will hopefully result in a network of young radiologist who have attended the Youth Club. The 2019 AOSOR Youth Club is taking place October 16-19 in Nagoya, Japan.

### The situation in Australia and New Zealand

L. Lawler presented on behalf of the **Royal Australian and New Zealand College of Radiologists (RANZCR)**. A large part of the RANZCR responsibilities is to set the curriculum and administer training programmes for admission into radiology professions, accreditation for overseas-trained specialists, and a continuing professional development programme for the members. Speaking about the numbers, L. Lawler pointed out that the RANZCR currently has a 1:4 ratio between students and practicing radiologists. Apart from the training programme itself, the RANZCR connects with trainees at every level through elected representative trainee committees as well as by having trainee representative on the Council and most decision-making committees. A Trainee Learning Day for clinical radiology and radiation oncology forms part of the College’s Annual Scientific Meeting (ASM). The Day’s program is determined by the Trainee Committees and usually includes teaching sessions, trainee scientific presentations, a discussion forum and dedicated topical sessions. An important element is the RANZCR Branch of Origin, established to support the training research requirements of Clinical Radiology. Participating Branches hold an annual trainee presentation evening with the most outstanding presentation from each Branch being presented at the College’s ASM in the ‘Branch of Origin’ session. The winner is announced at the Gala Dinner. Finally, L. Lawler pointed out that a big focus of the College staff is to maintain a social media presence as well.

### The situation in Europe

C. Catalano presented on behalf of the **European Society of Radiology (ESR)**. The main objectives of the ESR are to engage medical students early and throughout their medical school curricula as well as to emphasise the intellectual, imaging and procedural components of radiology. The ESR has produced the undergraduate curriculum, that is being updated every year, and has defined several aspects that need to be considered regarding the needs of the undergraduates. C. Catalano pointed out that the ESR pays a lot of attention to teachers, stressing the importance of providing adequate teaching materials. It was further stated that the AI is an enormous resource for radiology, but that it is also important to introduce the need for patient-centred radiology with A.I.’s active and increasing role in precision medicine. Over the last 16 years, the ESR has invited more than 8,000 young residents in radiology and radiographers in training to the European Congress of Radiology and continued developing its flagship youth project. Successful applicants for the Invest in the Youth programme were offered free registration for ECR and an accommodation voucher. C. Catalano mentioned other important ESR initiatives including “Coffee and talk” sessions dedicated to residents and trainees, as well as “Present my thesis in 3 minutes” aimed at further engaging young people who visit the conference. C. Catalano concluded that a bright future for radiology can only be ensured by recruiting those individuals that have the greatest likelihood of achieving job satisfaction in radiology.

### Position of the International Society of Radiology (ISR)

L. Donoso, on behalf of the **International Society of Radiology (ISR)**, presented the ISR findings regarding undergraduate radiology teaching from the student’s perspective. Radiology teaching should be represented in all pre-clinical and clinical undergraduate years. Medical students rate interactive case-based teaching sessions as very effective and there is a call for reliable, up-to-date open access electronic resources for medical students. L. Donoso also pointed out the importance of the “radiologist’s image”. Radiologists are no longer relegated to the back room where they interpret studies in a vacuum. Frequently clinician colleagues come to the reading room for radiologist’s opinions, and residents are an integral part of that process. Residents are often on the front lines, rendering opinions and discussing with clinicians how to manage particular patients. L. Donoso explained that the cross-generational teamwork in radiology can be easily fostered by involving millennials on tumour boards and in multidisciplinary conferences, especially those that emphasise the importance of imaging and image-guided procedures as part of the patient’s clinical care. When thinking about radiologists’ collective reputation patients’ opinion, referring physicians’ opinion and the opinion of the hospital need to be considered. L. Donoso further explained the need for the activities of the ISR stating that radiology needs a unified voice in dealing with global issues affecting radiology and the patients served. The World Health Organisation (WHO) and the International Atomic Energy Agency (IAEA) exert tremendous influence through regional and international initiatives on the appropriate use of medical radiation to improve population health. The ISR, in turn, serves as a primary advisor to the WHO and the IAEA in dealing with global issues affecting radiology. The main goals of the ISR are to promote global radiological quality and safety and bring high quality radiological education to underserved areas, acting as a convener and facilitator of content rather than the creator. Finally, L. Donoso mentioned specialised ISR programmes, Global Outreach: Education for Radiology (GOED) and Global Outreach Radiology (GORAD), that aggregate and disseminate high quality educational material from major journals and organisations to resource challenged areas.

## Conclusion

The current young generation is different than any generation that preceded them. Accordingly, the societies need to adapt their approach and adopt new strategies tailored specifically for the new generation in order to engage young doctors into radiology and ensure an increase in their membership base.

The societies understand that, in order to achieve these goals, it is important to first fully comprehend the character of the younger generation. In that sense, the CIR and the SMRI emphasise the importance of properly defining the millennials while the RSNA has developed a variety of tools aimed at understanding what young people want and need and what their expectations are. In Thailand, the societies developed unique approaches towards undergraduates and postgraduates, as it is important to understand the different needs these two groups might have. China is striving to create the best possible environment for the young generation and to develop personalised pathways to find a best possible match between an open position and an individual.

The ISR points out that, in order to engage the younger generation, it is also important to adapt the current image of a radiologist, and, in a similar vein, Colombia is focusing on mental barriers the radiologists themselves might have and is looking for ways of overcoming them.

A very important tool for understanding the younger generation as well as adapting the current image of the radiologist is the use of technology and social media and many societies are increasingly involving their current and future members in this way. The ACR has developed an internal networking platform called Engage. In Korea, the KSR is carefully listening to the needs expressed by the young radiologists and is using data analysis to develop the best approach based on the expressed needs. For Japan, understanding the technology and the A.I. is crucial for the engagement of the younger generation. The A.I. must be seen as an opportunity for radiology rather than a threat and public promotion is very important in that regard.

Delegating responsibilities and motivating residents are important aspects of strategies implemented by many societies. The CAR has accordingly implemented a strategy targeted specifically at residents and the RSNA gives special attention to early-career practitioners.

Perhaps the most important tool for further engaging the young generation is the use of innovative teaching solutions and increased participation in clubs, activities and other youth-oriented programmes. The IRIA has developed a specialised youth-oriented program (YUWA) working exclusively on the welfare of postgraduate residents and young radiologists. As it is important to engage younger generation in the actives early on, the AOSR is maintaining different clubs such as the AOSOR Youth Club, while the RANZCR has trainee representatives on the Council and most decision-making committees and organises events on topics decided by the trainees themselves. The FMRI is actively involving students in the learning process by including radiology in the University programmes and offering students with the best results the opportunity to become instructors after successfully finishing a course, thus giving the students an active role in the teaching-learning process. The SPR also uses innovative teaching solutions as well as increased participations in clubs and activities. Egypt is developing strategies to tackle concrete issues and create an approach that will benefit young generations in the entire country, while the CBR offers mobile learning initiatives and other educational activities. In Europe, the ESR is further developing its flagship youth project and has, over the last 16 years, invited more than 8,000 young residents in radiology and radiographers in training to participate in the European Congress of Radiology and its various events. The ESR has produced the undergraduate curriculum and is paying attention to the education of teachers as well.

Finally, many societies stress the importance of trainings abroad and appropriate fellowship programmes. In that sense, Saudi Arabia is placing great emphasis on fellowship trainings, especially those abroad, and the RSSA is playing an active role in education of younger generation of radiologists while the JRS is fostering exchange programmes such as the Japanese-German Radiological Affiliation and the French Society of Radiology – Japan Radiological Society (SFR-JRS) Fellowship Program Exchange.

Radiology is changing; in parallel to these changes a new approach towards the younger generation needs to be adopted. The technology plays a key role in this process and will serve as a bridge between already established and future radiologists, thus ensuring a bright future for radiology as a whole.

## Data Availability

All data generated or analysed during this study are included in this published article.

